# The Mechano-Genomic Frontier: Orchestrating Nuclear Deformation for Craniomaxillofacial Bone Regeneration

**DOI:** 10.3390/jcm15166191

**Published:** 2026-08-10

**Authors:** Caris M. Smith, Shawn A. Hallett, Jeremie O. Piña

**Affiliations:** 1 Department of Oral and Maxillofacial Surgery, School of Dentistry, University of Alabama at Birmingham, Birmingham, AL 35294, USA; csmith98@uab.edu; 2 Lumiere Education, Wilmington, DE 19801, USA; sahallett20@gmail.com; 3 Division of Oral and Maxillofacial Surgery, Department of Surgery, Emory University School of Medicine, Atlanta, GA 30322, USA

**Keywords:** craniomaxillofacial surgery, skull reconstruction, tissue engineering, nucleomechanical, nuclear deformation, cytoskeleton, 3-dimensional matrix, mesenchymal stem cell, growth factors

## Abstract

The paradigm of craniomaxillofacial (CMF) reconstruction is shifting from traditional bone grafting and biochemical adjuncts toward a nucleomechanical framework that leverages the cell nucleus as a mechanosensitive organelle. By utilizing computer-aided design and computer-aided manufacturing (CAD/CAM)-derived scaffolds with 10 µm micropillar arrays and specific interfacial stiffness (25–40 kPa), surgeons can physically manipulate the Linker of Nucleoskeleton and Cytoskeleton (LINC) complex to achieve a nuclear aspect ratio above 2.5. This structural deformation mechanically expands nuclear pores to trigger cytoskeletal and molecular responses, such as Yes-associated protein (YAP) and transcriptional coactivator with PDZ-binding motif (TAZ) translocation. Resultantly, this physical tension pulls open chromatin fibers to activate master osteogenic regulators like RUNX2, effectively bypassing the risks and limitations associated with supraphysiologic growth factor delivery (e.g., rhBMP-2). Clinically, translating these principles involves moving away from absolute rigid internal fixation toward advanced resorbable biomaterials that permit controlled micro-motions (100–200 µm) under functional masticatory loads. This review provides a structured synthesis of the field, outlining deterministic topographic criteria, clinical boundary conditions, and the potential strategies needed to overcome age-related mechanosensory blockades. Ultimately, we establish a multidisciplinary framework that bridges precision bioengineering with native oral and maxillofacial surgical realities to drive living, biophysically mediated bone repair.

## 1. Scope, Objectives, and Narrative Review Methodology

The clinical mandate for craniomaxillofacial (CMF) reconstruction focuses on treating critical-sized osseous defects resulting from ablative tumor surgery, severe trauma, or extensive congenital anomalies [1]. Within Oral and Maxillofacial Surgery (OMFS), these defects present a major physiological cliff where the localized metabolic demand outpaces the native supply of osteoprogenitor cells, causing substantial reconstructive hurdles [2]. While vascularized autologous bone grafts remain the clinical gold standard, their use is fundamentally limited by donor-site morbidity, anatomical volume constraints, and prolonged operative times that elevate systemic risk for elderly or medically compromised individuals [3].

For decades, the bioengineering field has sought a synthetic equivalent by combining passive scaffold matrices, progenitor cells, and exogenous growth factors like recombinant human bone morphogenetic protein-2 (rhBMP-2) [4]. However, rhBMP-2 application within the craniofacial skeleton remains highly controversial due to its supra-physiological dosing requirements [5,6]. Local delivery is frequently complicated by rapid therapeutic washout, poor craniofacial skeletal penetration, and severe local adverse effects, including life-threatening cervical edema, heterotopic ossification, and intense localized inflammation near critical structures like the airway or optic nerve [7,8,9].

Consequently, the field is transitioning toward exploiting the intrinsic biophysical properties that govern skeletal development, repair, and tissue homeostasis [10,11]. Rather than treating a scaffold as a passive pharmaceutical reservoir, modern material-driven differentiation focuses on utilizing precisely engineered surface topographies and mechanical gradients to directly guide cellular fate [12,13]. While historical research focused on cell-membrane integrins as the primary mechanosensors, emerging nucleomechanical literature reveals that the nucleus itself acts as a central governor that directly integrates external physical forces [14,15]. By utilizing functional masticatory forces, complex torsion, and shear loads unique to the mandibular niche [16,17], custom scaffolds can physically deform the cell nucleus to directly unlock osteogenic gene networks without pharmaceutical dependency.

### 1.1. Core Review Questions

To provide a disciplined exploration of this emerging field, this narrative review is strictly structured around two central, verifiable scientific questions:

1. How do the physical configurations of micro-topographic scaffold interfaces dictate nuclear geometry to initiate cell-autonomous osteogenesis without external growth factors?
2. How can mechanical loading from the functional masticatory cycle be structurally translated into regenerative dosing while maintaining biomechanical safety boundaries in the surgical site?

### 1.2. Literature Selection and Search Strategy

A systematic search of literature published between January 2000 and May 2026 was conducted using the PubMed, Embase, and Web of Science databases. Keywords utilized in combination included: “craniomaxillofacial reconstruction,” “nuclear deformation,” “nucleomechanics,” “LINC complex,” “mechanotransduction,” “deterministic topographies,” and “mandibular micro-motion.”

Inclusion Criteria: Peer-reviewed experimental studies, clinical trials, and technical reviews that evaluated biophysical force transmission, nuclear structural changes, material-driven stem cell differentiation, or finite element analysis of CMF implants.

Exclusion Criteria: Articles evaluating purely biochemical or pharmacological delivery systems lacking structural, topographical, or mechanical characterization, and abstracts or conference proceedings without full text.

Evidence Grading: Identified literature was classified according to standard evidence hierarchies: Level I, which represents randomized controlled trials or systematic reviews; Level II, which represents well-designed cohort or controlled laboratory studies; and Level III, which represents descriptive laboratory investigations, 2D in vitro cell assays, and expert opinions. Throughout this review, hypotheses and laboratory findings are explicitly demarcated to separate established in vivo realities from conceptual medical frameworks.

## 2. The Nucleus as a Mechanical Gateway to Genetic Alterations

Historically viewed as a passive, protected genomic repository, the nucleus is now recognized as a dynamic, highly connected mechanosensitive organelle that is physically linked to the extracellular matrix [18]. Biophysical micro-indentation and atomic force microscopy have established that the nucleus is 2 to 10 times stiffer than the surrounding cytoplasm, serving as the cell’s primary mechanostat [19,20]. This structural coupling is mediated by the Linker of Nucleoskeleton and Cytoskeleton (LINC) complex [21]. Nesprin proteins embedded in the outer nuclear membrane project Klarsicht, ANC-1, Syne homology (KASH) domains into the perinuclear space to bind Sad1 and UNC-84 (SUN) proteins. These SUN proteins traverse the inner nuclear membrane to anchor directly to the dense nuclear lamina composed of Lamin A/C and B. When mesenchymal stem cells (MSCs) spread across an engineered substrate, actomyosin contractility pulls directly on this SUN-KASH molecular bridge, transmitting physical tension straight to the nuclear envelope [20,22].

### 2.1. Confirmed Biophysical Findings: Nuclear Pore Dilation and Epigenetic Opening

**Figure 1:**
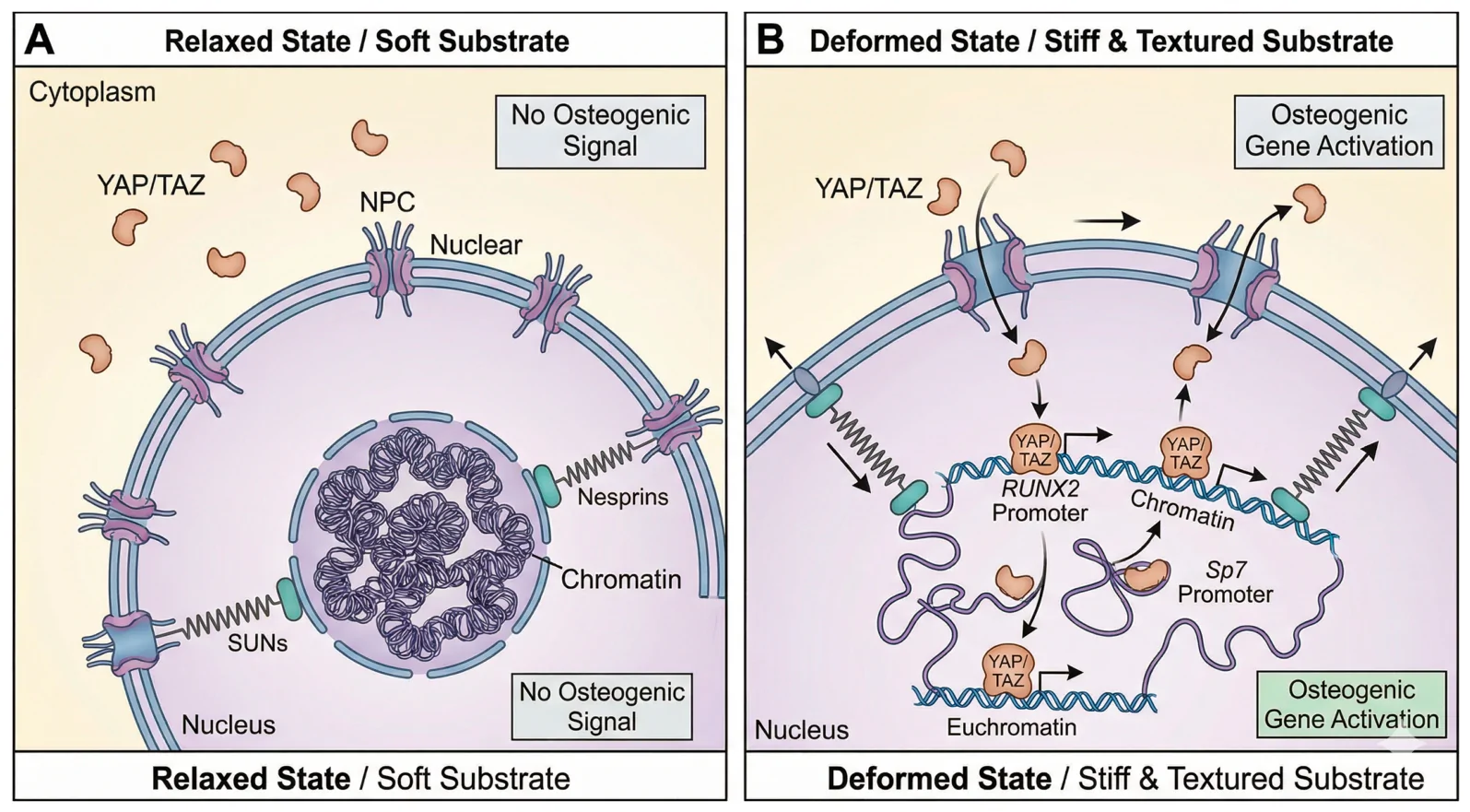
Molecular mechanism of mechanotransduction. (**A**) On compliant substrates, low cytoskeletal tension maintains a spherical nucleus with constricted nuclear pore complexes (NPCs), sequestering YAP/TAZ in the cytoplasm. (**B**) On rigid, micro-patterned surfaces, force transmitted via the LINC complex physically dilates NPCs. This dilation facilitates the nuclear translocation of YAP/TAZ, promoting chromatin remodeling and the subsequent upregulation of master osteogenic regulators, including RUNX2 and SP7. All schematic elements are original illustrations generated by the authors, aided by Python coding (available upon request).

In vitro biophysical models demonstrate that cells adhering to rigid, highly spread surfaces (30–40 kPa) experience substantial nuclear strain and envelope wrinkling compared to those on soft substrates (1–5 kPa) [23,24]. This physical strain deforms the nuclear envelope, resulting in the direct expansion of Nuclear Pore Complexes (NPCs) [25,26]. High-resolution cryo-electron tomography shows that stretching the nuclear lamina reduces the local curvature of the NPC scaffold, physically dilating the central transport channel [27]. In a relaxed or spherical cell state, these channels restrict the transport of macromolecular transcription factors. Upon mechanical flattening or elongation, the expanded NPC diameter allows rapid, passive diffusion of Yes-associated protein (YAP) and transcriptional coactivator with PDZ-binding motif (TAZ) from the cytoplasm into the nucleus, operating at a velocity that rivals active Ran-GTPase transport [25]. Once inside, YAP/TAZ complex with the TEA domain (TEAD) family of transcription factors to drive the primary osteogenic program, upregulating Runt-related transcription factor 2 (RUNX2) and Sp7 (Osterix) [28,29] (Figure 1).

Simultaneously, nuclear deformation induces critical epigenetic shifts by physically manipulating chromatin architecture [30]. Densely packed heterochromatin is naturally localized at the nuclear periphery, tethered to lamina-associated domains. Mechanical strain on the polymer meshwork of A-type and B-type lamins triggers the physical dissociation of heterochromatin-silencing proteins, transforming transcriptionally inactive heterochromatin into open, accessible euchromatin [31,32]. Studies utilizing Assay for Transposase-Accessible Chromatin (ATAC) verify that MSCs undergoing controlled nuclear deformation exhibit rapid chromatin opening at the promoter loci for Alkaline Phosphatase (ALP) and Osteocalcin (OC) within 2 to 4 h of physical stimulus, entirely bypassing the need for exogenous growth factors [33].

Furthermore, this nuclear strain activates a pro-regenerative matricrine secretome [34]. Cells experiencing topographical deformation alter their intracellular state and upregulate the secretion of extracellular vesicles, Type I Collagen (Col1), and Vascular Endothelial Growth Factor (VEGF) [35]. Recent micro-patterned studies demonstrated that 10 µm micropillar-induced deformation led to a 3.4-fold increase in the release of pro-osteogenic exosomal microRNAs (e.g., miR-21), accelerating mineralization in neighboring host cells not in direct contact with the substrate [34]. This paracrine cascade allows a localized micro-topography to exert a broader regenerative influence on the surrounding host tissue bed.

### 2.2. Translational Concepts: Overcoming the Mechanosensory Blockade in Elderly Patients

While these biophysical mechanisms are well-documented in healthy cell lines, translating them into the primary clinical target demographic—elderly patients requiring bone plastic surgery—presents a major hurdle. In aging MSCs, a shifting ratio of Lamin A to Lamin C and the gradual accumulation of progerin-like truncated lamins result in rigid nucleus syndrome [20,36,37]. This age-related nuclear stiffening acts as a severe mechanosensory blockade; the rigid lamina prevents the physical expansion of NPCs and the subsequent mechanical opening of chromatin loci under standard loads, explaining the diminished regenerative capacity and increased incidence of osteoporotic density loss in older individuals [38].

To overcome this age-related barrier and restore biophysical responsiveness, this review highlights three potential therapeutic strategies currently under active investigation:

1. Epigenetic Priming via HDAC Inhibition: Administering low-dose, localized histone deacetylase (HDAC) inhibitors can manually de-condense highly packed heterochromatin, reducing the mechanical threshold of force required to expose bone-specific gene promoters in rigid nuclei.
2. Cytoskeletal Modulation: Temporary biochemical stabilization of actin-myosin contractility (e.g., via small-molecule activators of RhoA signaling) can boost intracellular traction forces, providing the necessary mechanical leverage to deform a stiffer, aging nuclear envelope.
3. Hyper-Dense Topographical Engineering: Redesigning scaffold surfaces with higher spatial frequencies and taller micropillar topographies to deliberately optimize focal adhesion placement, maximizing the physical torque applied across the LINC complex of senescent cells.

## 3. Engineering the Interface: Designing for Controlled Deformation

From a precision bioengineering standpoint, generating reliable osteogenic commands requires the implementation of deterministic topographies that eliminate stochastic cellular behavior [39]. Deterministic surfaces utilize micro-scale features—such as pillars, grooves, or channels—to force the infiltrating cell nucleus into a highly predictable geometric conformation. These surfaces operate on the mathematical principle of spatial frequency, where the density and arrangement of features must precisely match the intrinsic length scale and contractility of the cell’s actin filaments [40].

### 3.1. The Pitch Paradox and Geometric Criteria

The primary design challenge for CMF scaffolds is managing the pitch paradox [41]. If topographical features are positioned too close together (pitch < 2 µm), the cell perceives the substrate as a flat surface, undergoing isostatic spreading that allows the nucleus to remain un-deformed and spherical [42]. Conversely, if features are too far apart, cells fail to bridge the gaps, entering a state of mechanical suspension characterized by a lack of focal adhesion kinase activation and subsequent adipogenic rather than osteogenic commitment [43].

Extensive laboratory investigations demonstrate that the optimal micro-architecture for driving osteogenesis consists of micropillars with a diameter of 10 µm, a height of 10 µm, and an inter-feature spacing (pitch) of 10–15 µm [44,45]. This specific configuration forces the cell body to bridge across adjacent pillars, maximizing the Nuclear Aspect Ratio (NAR), which is mathematically defined as the ratio of the major axis to the minor axis:

NAR = a/b

Recent quantitative metrics indicate that achieving an NAR > 2.5 represents the critical biophysical threshold required to dilate NPCs and induce cell-autonomous osteogenesis [46].

### 3.2. Viscoelastic Modeling and Finite Element Analysis (FEA)

While linear elastic theory models structural strain energy density as U = 0.5 * E * ε^2^, the cell-nucleus interface behaves as a viscoelastic Kelvin-Voigt model, meaning that load timing and frequency are as critical as force magnitude [47]. To optimize this, the scaffold must feature a distinct stiffness gradient: while the bulk modulus must remain rigid enough to withstand macro-scale jaw forces (15–20 GPa), the interfacial contact surface must be engineered to be bio-soft (25–40 kPa) [48,49]. If the contact interface is too stiff, the LINC complex proteins reach their mechanical rupture force before meaningful nuclear deformation occurs. Maintaining an interfacial stress (σ = E * ε) within this bio-soft window ensures that typical physiological loading translates into a safe 5–12% local cellular strain, delivering approximately 15 pN of force per Nesprin-1 protein to safely flatten the nucleus [50,51].

Multiscale Finite Element Analysis (FEA) has become essential to map these force distributions across complex geometries [52]. High-resolution digital renderings of patient-specific defects allow engineers to simulate multi-vector masticatory force cycles using the Von Mises stress criterion, successfully identifying and eliminating mechanical “dead zones” (stress < 1 MPa) that could lead to constructive graft resorption [53]. Modern multiscale FEA routines can simulate individual cell-scaffold contacts across an entire 5 cm mandibular segment, predicting the precise NAR for over 100,000 cells simultaneously [54]. This uniform strain field directly addresses the historical boundary effect gap, ensuring that stem cells located in the deep ischemic core of the scaffold receive the same osteogenic mechanical command as those situated at the well-vascularized host-graft interface [55].

A major unknown remaining in this domain is the long-term impact of viscoelastic creep and material degradation [56,57]. Biodegradable 3D-printed polymers, such as polycaprolactone (PCL), exhibit a loss of stiffness over time that follows a pseudo-first-order kinetic decay model:

Et = E0 * e^(−kt)

If material degradation outpaces new bone deposition, the essential nuclear deformation signal is lost prematurely [58]. To resolve this, 4D bioprinting and shape-memory polymers are being deployed to program a time-dependent increase in scaffold rigidity that mirrors the peak of the MSC-to-osteoblast transition, maintaining a constant deformation pressure throughout the tissue-healing timeline [59,60,61,62].

Furthermore, fabricating these sub-micron deterministic topographies across large, macro-scale surgical volumes presents a severe precision-to-volume scalability bottleneck:

Rp = Resolution/Total Volume

**Figure 2:**
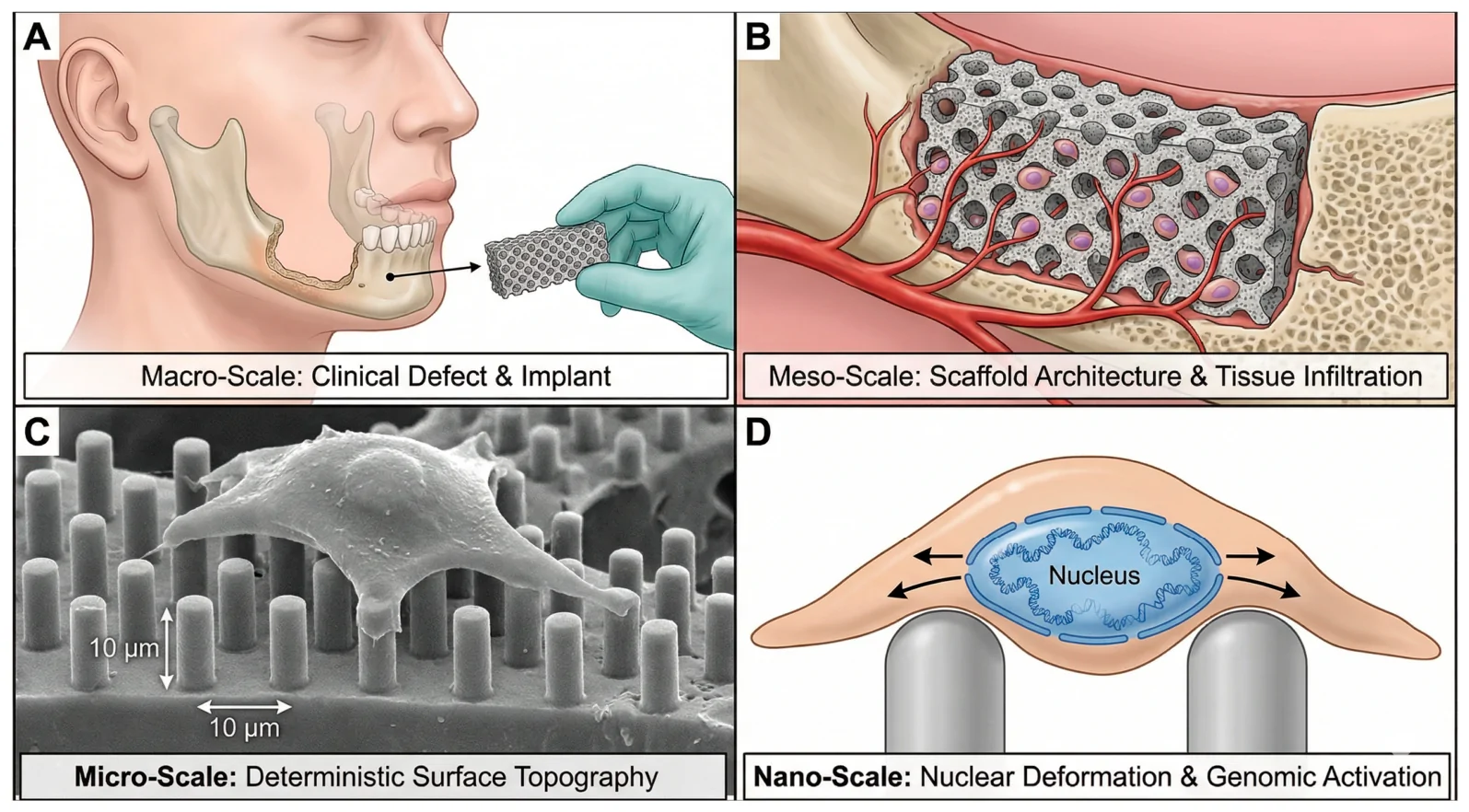
Harnessing nuclear deformation for craniomaxillofacial tissue regeneration. (**A**) Segmental mandibular defect receiving a patient-specific, 3D-printed bio-instructive scaffold. (**B**) Cellular infiltration and neovascularization within the scaffold lattice. (**C**) Adhesion of a mesenchymal stem cell (MSC) to a deterministic surface topography (10 µm pillar pitch). (**D**) Nano-scale deformation of the nucleus (NAR > 2.5) under functional masticatory loads, acting as the primary trigger for osteogenic lineage commitment. All schematic elements are original illustrations generated by the authors, aided by Python 3.14 coding (available upon request).

To overcome this, hybrid fabrication approaches are utilized, combining a high-strength, 3D-printed titanium core (providing the 15 GPa bulk modulus needed for immediate structural stability) coated with a precision-molded polymer sleeve engineered via two-photon polymerization to exhibit the exact 10 µm deterministic micropillar architecture [63]. This decouples bulk mechanical load-bearing from surface biological signaling, satisfying both the structural requirements of the surgeon and the biophysical needs of the regenerating cells (Figure 2).

## 4. The OMFS Surgical Reality: Harnessing the Masticatory Cycle

Translating these laboratory mechanobiological findings into clinical oral and maxillofacial surgery requires a careful re-examination of the rigid internal fixation (RIF) paradigm. Since the advent of modern AO principles in the 1970s, surgical mandate has prioritized absolute construct immobilization to prevent fibrous non-union [64]. However, absolute rigidity using thick titanium plates frequently causes severe stress shielding, where the hardware bears 100% of the mechanical load, leaving the underlying bone un-stimulated and highly susceptible to disuse atrophy and delayed mineralization [65,66]. This clinical challenge represents the RIF paradox, where the rigid methods traditionally employed to stabilize a defect inadvertently suppress the natural mechanotransduction cascades needed by native cells within the reconstructive niche [67,68].

### 4.1. Hypothesized Concepts and Translational Safety Boundaries

To bridge this gap, modern bioengineering frameworks propose replacing absolute rigid fixation with advanced resorbable plates (e.g., magnesium-based or customized copolymer hardware) designed to permit controlled micro-motions of 100–200 µm after a stable vascular bed is established around week 4 of healing [68,69].

Critical Translatability Note: It must be explicitly stated that the clinical implementation of controlled micro-motion (100–200 µm) within critical-sized mandibular defects remains an unverified hypothesis that requires rigorous in vivo validation. While small-animal appendicular models demonstrate that controlled micro-movements accelerate callus formation, large-animal craniomaxillofacial data confirming its safety without inducing fibrous encapsulation are highly limited. The mandibular complex is subject to complex multi-vector forces (torsion, shear, and tension) rather than pure axial compression. Consequently, if micro-movements are poorly controlled or introduced prematurely before adequate neo-vascularization, there is a high risk of mechanical instability leading to persistent fibrous non-union, construct failure, or micro-fracture at the host-graft interface. This approach must currently be treated as a highly promising mechanobiological concept requiring formal large-animal safety confirmation.

### 4.2. Surgical Management: Infection, Vascularization, and Digital Workflow

Beyond fixation, the surgical team must navigate the hostile, highly contaminated environment of the oral cavity. The persistent threat of salivary contamination and subsequent bacterial biofilm formation (e.g., by Porphyromonas gingivalis) on implanted hardware remains a primary cause of graft failure and chronic osteomyelitis [70]. A major historical failure mode of large bio-synthetic scaffolds is that the human immune system perceives the un-endothelialized implant as a foreign body, triggering an aggressive rejection or chronic inflammatory response [71,72]. Standard systemic antibiotic prophylaxis frequently fails to penetrate the ischemic center of a newly placed large-volume graft [73].

To address this challenge, surgical research is evaluating bifunctional smart hydrogel coatings [74]. These materials are engineered to be anti-adhesive for bacteria during the acute, low-pH inflammatory phase immediately following surgery. As the local microenvironment stabilizes and returns to a physiological pH, the hydrogel undergoes a predictable conformational change, revealing Arg-Gly-Asp (RGD) binding motifs that actively promote host osteoblast attachment, closing the sterile window and initiating biophysical force loops [74].

Furthermore, because neo-vascularization is the ultimate bottleneck in defects exceeding 6 cm, scaffold designs must incorporate pre-engineered vascular channels [75,76]. Seeding these pathways with autologous platelet-rich fibrin (PRF) or endothelial cells at the time of operation ensures that infiltrating osteoprogenitors do not succumb to ischemic hypoxia [77]. This creates a self-sustaining regenerative cycle: initial controlled physical loading drives deformed osteoblasts to secrete heightened levels of VEGF, which actively pulls neo-vasculature into the core of the scaffold, providing the persistent oxygenation required for sustained osteoblast function [78].

Finally, ensuring seamless force transmission across this multi-scale circuit requires absolute anatomic fit. Any gap exceeding a few microns at the host-graft junction acts as an absolute mechanical insulator, preventing the natural kinetic energy of the masticatory muscles from reaching the cellular nuclear sensors within the graft core [16]. This requires a rigid digital workflow incorporating Virtual Surgical Planning (VSP) and CAD/CAM technologies [79,80]. By utilizing high-resolution pre-operative CT scans, haptic feedback modeling, and custom 3D-printed cutting guides, surgeons can perform precise osteotomies that maximize the direct surface contact area against the native ramus or symphysis, effectively closing the mechanobiological circuit the moment the construct is seated [79].

Following surgery, traditional protocols that mandate a soft diet for 6–8 weeks are replaced with precise, progressive rehabilitation regimens [81]. Introducing a graduated mechanical loading dose—progressing from early soft-tissue manipulation to controlled, low-impact masticatory cycles—serves as a physical prescription that safely guides nuclear deformation, turning the patient’s own functional energy into a tool for permanent, living skeletal repair [81].

## 5. Synthesis Matrix: Structural Status of the Field

To maintain a strict division between established scientific consensus and emerging hypotheses (as mandated by standard review methodologies), the current state of the nucleomechanical frontier is summarized below, broken up by their biophysical category (italics), the confirmed in vitro/in vivo evidence (level II/III), as well as their hypothesized translational applications:

Nuclear Pore Activation: Cryo-EM and AFM confirm that nuclear flattening >2.5 NAR dilates NPCs, forcing passive YAP/TAZ nuclear translocation within 2 h [23,25,27]. Standardized mechanical loading prescriptions or physical exercise regimens can be tailored to human post-operative timelines [81].

Epigenetic Chromatin Opening: ATAC-seq confirms that physical strain on the lamin meshwork dissociates heterochromatin, opening ALP and OC promoter loci [31,33]. Pharmacological reversing of rigid nucleus syndrome in clinical human cohorts using localized epigenetic adjuvants has been demonstrated [20,36].

Topographic Specifications: In vitro validation of deterministic 10 µm micropillar arrays with a 10–15 µm pitch to optimize intracellular tension and cell fate [44,45]. Multi-scale FEA modeling predicts uniform 5–12% strain fields across large-scale, multi-axial human mandibular defects [53,54].

Fixation Dynamics: Conventional rigid internal fixation prevents disuse atrophy patterns in appendicular skeletons; small-animal models show micromotion utility [64,68]. Safe execution of 100–200 µm micro-motion resorbable plates in large-animal or human critical-sized CMF defects without fibrous union has been demonstrated [68,69].

### Translational Limitations and the In Vivo Dimensionality Gap

A primary limitation of the fundamental biophysical data compiled across the current literature is its heavy reliance on simplified, two-dimensional (2D) in vitro cell culture models. While 2D micro-patterned chips provide an excellent, high-resolution platform to observe isolated nuclear deformation, they fail to replicate the true dimensionality of a three-dimensional (3D) porous scaffold in vivo.

**Figure 3:**
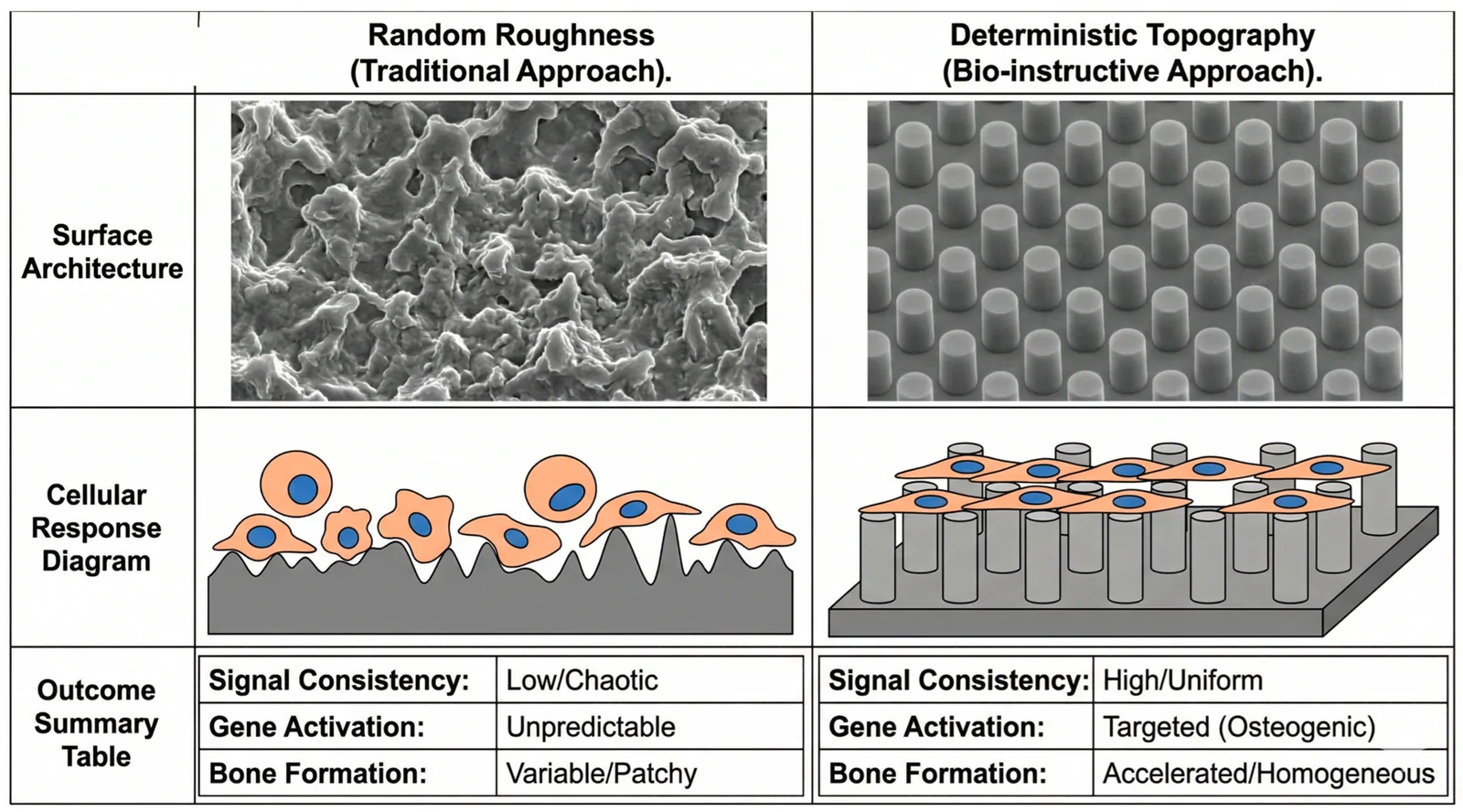
Comparison of random to deterministic topographies. (**Left**) Traditional random roughness yields stochastic cellular responses, inconsistent nuclear geometries, and unpredictable mineralized bone formation. (**Right**) Deterministic micropillar arrays provide uniform mechanical cues across the scaffold interface, ensuring predictable nuclear deformation and accelerated, homogeneous bone matrix deposition. All schematic elements are original illustrations generated by the authors, aided by Python coding (available upon request).

Within a living, critical-sized bone defect, infiltrating progenitor cells are not confined to a flat plane; they are embedded within a complex 3D architecture where they are exposed to simultaneous multiaxial forces, interstitial fluid shear stress, and dynamic cell-to-cell and cell-to-matrix interactions. Furthermore, the local physical microenvironment is constantly altered by matrix metalloproteinases (MMPs) and macrophage-mediated material degradation. Consequently, a surface topography that elicits a precise NAR > 2.5 in a static, isolated incubator environment may perform unpredictably when subjected to the dynamic, fluid-filled, multi-vector stress fields of an in vivo maxillofacial niche. This severe lack of high-fidelity 3D in vivo data represents the most critical barrier preventing the widespread translation of nucleomechanical engineering from the laboratory bench to the oral and maxillofacial operating theater (Figure 3).

## 6. Conclusions and Future Directions

The transition toward biophysically focused craniomaxillofacial reconstruction marks a significant departure from the pharmacological era of growth factor delivery. By treating the cell nucleus as a mechanical actuator directly coupled to the extracellular environment, bioengineers can leverage deterministic scaffold topographies to directly activate master osteogenic networks. To successfully translate these principles into predictable surgical outcomes, future research must move toward resolving the precision-to-volume fabrication bottleneck via hybrid multi-scale printing and deploying 4D shape-memory materials that maintain mechanical dosing throughout the graft-healing timeline. Most critically, extensive large-animal in vivo trials are urgently required to establish the precise safety boundaries of controlled micro-motion fixations, ensuring that functional masticatory loads can be safely harnessed to drive reliable, living bone regeneration without the risk of fibrous non-union.

## Data Availability

No new data were created or analyzed in this study. Data sharing is not applicable to this article.

## Abbreviations

The following abbreviations are used in this manuscript:

CMF	Craniomaxillofacial
OMFS	Oral and Maxillofacial Surgery
rhBMP-2	Bone Morphogenetic Protein 2
MSC	Mesenchymal Stem Cell
LINC	Linker of Nucleoskeleton and Cytoskeleton
SUN	Sad1 and UNC-84
KASH	Klarsicht, ANC-1, Syne homology
NPC	Nuclear Pore Complex
YES	Yes-associated protein
PDZ	PSD95, DlgA and zonula occludens-1 protein
TAZ	Transcriptional coactivator with PDZ-binding motif
TEAD	TEA domain
RUNX2	Runt-related transcription factor 2
Sp7	Osterix
VEGF	Vascular endothelial growth factor
NAR	Nuclear Aspect Ratio
FEA	Finite Element Analysis
PEEK	Polyetheretherketone
PCL	Polycaprolactone
RIF	Rigid Internal Fixation
AO	Arbeitsgemeinschaft für Osteosynthesefragen
MMP	Matrix metalloproteinases

## References

[1] Nayak V.V., Slavin B.V., Bergamo E.T., Torroni A., Runyan C.M., Flores R.L., Kasper F.K., Young S., Coelho P.G., Witek L. (2023). Three-Dimensional Printing Bioceramic Scaffolds Using Direct-Ink-Writing for Craniomaxillofacial Bone Regeneration. Tissue Eng. Part C Methods.

[2] Zhang C., Chen X., Ying W., Zhang C. (2025). Global burden of plastic-surgery-related conditions, 1990-2021: A composition-aware analysis with projections to 2050. Front. Public Health.

[3] Chenard K.E., Teven C.M., He T.C., Reid R.R. (2012). Bone morphogenetic proteins in craniofacial surgery: Current techniques, clinical experiences, and the future of personalized stem cell therapy. J. BioMed Biotechnol..

[4] Dixon D.T., Gomillion C.T. (2021). Conductive Scaffolds for Bone Tissue Engineering: Current State and Future Outlook. J. Funct. Biomater..

[5] Bouyer M., Guillot R., Lavaud J., Plettinx C., Olivier C., Curry V., Boutonnat J., Coll J.-L., Peyrin F., Josserand V. (2016). Surface delivery of tunable doses of BMP-2 from an adaptable polymeric scaffold induces volumetric bone regeneration. Biomaterials.

[6] Zeng Y., Hoque J., Varghese S. (2019). Biomaterial-assisted local and systemic delivery of bioactive agents for bone repair. Acta Biomater..

[7] Hollister S.J., Murphy W.L. (2011). Scaffold translation: Barriers between concept and clinic. Tissue Eng. Part B Rev..

[8] Raghavan M., Ong A.A., Carr M.M. (2025). Complications After Craniofacial Surgery: A Review From 2012 to 2020. Cureus.

[9] Hong L., Sun H., Amendt B.A. (2021). MicroRNA function in craniofacial bone formation, regeneration and repair. Bone.

[10] Alikhani M., Alikhani M., Alansari S., Almansour A., Hamidaddin M.A., Khoo E., Lopez J.A., Nervina J.M., Nho J.Y., Oliveira S.M. (2019). Therapeutic effect of localized vibration on alveolar bone of osteoporotic rats. PLoS ONE.

[11] Florencio-Silva R., Sasso G.R., Sasso G.R.D.S., Simões M.J., Cerri P.S. (2015). Biology of Bone Tissue: Structure, Function, and Factors That Influence Bone Cells. BioMed Res. Int..

[12] Cun X., Hosta-Rigau L. (2020). Topography: A Biophysical Approach to Direct the Fate of Mesenchymal Stem Cells in Tissue Engineering Applications. Nanomaterials.

[13] Donnelly H., Smith C.-A., Sweeten P.E., Gadegaard N., Meek R.D., D’eSte M., Mata A., Eglin D., Dalby M.J. (2017). Bone and cartilage differentiation of a single stem cell population driven by material interface. J. Tissue Eng..

[14] Dahl K.N., Ribeiro A.J.S., Lammerding J. (2008). Nuclear shape, mechanics, and mechanotransduction. Circ. Res..

[15] Niethammer P. (2021). Components and Mechanisms of Nuclear Mechanotransduction. Annu. Rev. Cell Dev. Biol..

[16] Clark J.R., Al Maruf D.S.A., Tomaskovic-Crook E., Cheng K., Lewin W.T., Xin H., Wan B., Ren J., Wu C., Kruse H.V. (2025). Mechanobiologically-optimized non-resorbable artificial bone for patient-matched scaffold-guided bone regeneration. Nat. Commun..

[17] Van Eijden T.M. (2000). Biomechanics of the mandible. Crit. Rev. Oral Biol. Med..

[18] Kalukula Y., Stephens A.D., Lammerding J., Gabriele S. (2022). Mechanics and functional consequences of nuclear deformations. Nat. Rev. Mol. Cell Biol..

[19] Dickinson R.B., Neelam S., Lele T.P. (2015). Dynamic, mechanical integration between nucleus and cell- where physics meets biology. Nucleus.

[20] Heo S.-J., Driscoll T.P., Thorpe S.D., Nerurkar N.L., Baker B.M., Yang M.T., Chen C.S., Lee D.A., Mauck R.L. (2016). Differentiation alters stem cell nuclear architecture, mechanics, and mechano-sensitivity. eLife.

[21] Crisp M., Liu Q., Roux K., Rattner J., Shanahan C., Burke B., Stahl P.D., Hodzic D. (2006). Coupling of the nucleus and cytoplasm: Role of the LINC complex. J. Cell Biol..

[22] Maurer M., Lammerding J. (2019). The Driving Force: Nuclear Mechanotransduction in Cellular Function, Fate, and Disease. Annu. Rev. BioMed Eng..

[23] Buxboim A., Irianto J., Swift J., Athirasala A., Shin J.-W., Rehfeldt F., Discher D.E. (2017). Coordinated increase of nuclear tension and lamin-A with matrix stiffness outcompetes lamin-B receptor that favors soft tissue phenotypes. Mol. Biol. Cell.

[24] Smith L., Cho S., Discher D.E. (2017). Mechanosensing of matrix by stem cells: From matrix heterogeneity, contractility, and the nucleus in pore-migration to cardiogenesis and muscle stem cells in vivo. Semin. Cell Dev. Biol..

[25] Elosegui-Artola A., Andreu I., Beedle A.E.M., Lezamiz A., Uroz M., Kosmalska A.J., Oria R., Kechagia J.Z., Rico-Lastres P., Le Roux A.-L. (2017). Force Triggers YAP Nuclear Entry by Regulating Transport across Nuclear Pores. Cell.

[26] Scott K.E., Fraley S.I., Rangamani P. (2021). A spatial model of YAP/TAZ signaling reveals how stiffness, dimensionality, and shape contribute to emergent outcomes. Proc. Natl. Acad. Sci. USA.

[27] Cardarelli F., Lanzano L., Gratton E. (2012). Capturing directed molecular motion in the nuclear pore complex of live cells. Proc. Natl. Acad. Sci. USA.

[28] Van Soldt B.J., Cardoso W.V. (2020). Hippo-Yap/Taz signaling: Complex network interactions and impact in epithelial cell behavior. Wiley Interdiscip. Rev. Dev. Biol..

[29] Zhao X., Tang L., Le T.P., Nguyen B.H., Chen W., Zheng M., Yamaguchi H., Dawson B., You S., Martinez-Traverso I.M. (2022). Yap and Taz promote osteogenesis and prevent chondrogenesis in neural crest cells in vitro and in vivo. Sci. Signal.

[30] Miroshnikova Y.A., Nava M.M., Wickström S.A. (2017). Emerging roles of mechanical forces in chromatin regulation. J. Cell Sci..

[31] Tajik A., Zhang Y., Wei F., Sun J., Jia Q., Zhou W., Singh R., Khanna N., Belmont A.S., Wang N. (2016). Transcription upregulation via force-induced direct stretching of chromatin. Nat. Mater..

[32] Thorpe S.D., Lee D.A. (2017). Dynamic regulation of nuclear architecture and mechanics-a rheostatic role for the nucleus in tailoring cellular mechanosensitivity. Nucleus.

[33] Zhang K., Qiu W., Li H., Li J., Wang P., Chen Z., Lin X., Qian A. (2023). MACF1 overexpression in BMSCs alleviates senile osteoporosis in mice through TCF4/miR-335-5p signaling pathway. J. Orthop. Transl..

[34] Wang X., Li Y., Lin Z., Pla I., Gajjela R., Mattamana B.B., Joshi M., Liu Y., Wang H., Zun A.B. (2025). Microtopography-induced changes in cell nucleus morphology enhance bone regeneration by modulating the cellular secretome. Nat. Commun..

[35] Paiva K.B.S., Maas C.S., Dos Santos P.M., Granjeiro J.M., Letra A. (2019). Extracellular Matrix Composition and Remodeling: Current Perspectives on Secondary Palate Formation, Cleft Lip/Palate, and Palatal Reconstruction. Front. Cell Dev. Biol..

[36] Afilalo J., Sebag I.A., Chalifour L.E., Rivas D., Akter R., Sharma K., Duque G. (2007). Age-related changes in lamin A/C expression in cardiomyocytes. Am. J. Physiol. Heart Circ. Physiol..

[37] Alcorta-Sevillano N., Macías I., Rodríguez C.I., Infante A. (2020). Crucial Role of Lamin A/C in the Migration and Differentiation of MSCs in Bone. Cells.

[38] Inoue M., Ono T., Kameo Y., Sasaki F., Ono T., Adachi T., Nakashima T. (2019). Forceful mastication activates osteocytes and builds a stout jawbone. Sci. Rep..

[39] Nakanishi J., Yamamoto S. (2022). Static and photoresponsive dynamic materials to dissect physical regulation of cellular functions. Biomater. Sci..

[40] Miyoshi H., Adachi T. (2014). Topography design concept of a tissue engineering scaffold for controlling cell function and fate through actin cytoskeletal modulation. Tissue Eng. Part B Rev..

[41] Dang H.P., Shabab T., Shafiee A., Peiffer Q.C., Fox K., Tran N., Dargaville T.R., Hutmacher D.W., Tran P. (2019). 3D printed dual macro-, microscale porous network as a tissue engineering scaffold with drug delivering function. Biofabrication.

[42] De Peppo G.M., Agheli H., Karlsson C., Ekstrom K., Brisby H., Lenneras M., Gustafsson S., Sjövall P., Johansson A., Olsson E. (2014). Osteogenic response of human mesenchymal stem cells to well-defined nanoscale topography in vitro. Int. J. Nanomed..

[43] Mathieu P.S., Loboa E.G. (2012). Cytoskeletal and focal adhesion influences on mesenchymal stem cell shape, mechanical properties, and differentiation down osteogenic, adipogenic, and chondrogenic pathways. Tissue Eng. Part B Rev..

[44] Bao M., Xie J., Piruska A., Huck W.T.S. (2017). 3D microniches reveal the importance of cell size and shape. Nat. Commun..

[45] Hasturk O., Ermis M., Demirci U., Hasirci N., Hasirci V. (2019). Square prism micropillars on poly(methyl methacrylate) surfaces modulate the morphology and differentiation of human dental pulp mesenchymal stem cells. Colloids Surf. B Biointerfaces.

[46] Taniguchi R., Orniacki C., Kreysing J.P., Zila V., Zimmerli C.E., Böhm S., Turoňová B., Kräusslich H.-G., Doye V., Beck M. (2025). Nuclear pores safeguard the integrity of the nuclear envelope. Nat. Cell Biol..

[47] Chojowski R., Schwarz U.S., Ziebert F. (2024). The role of the nucleus for cell mechanics: An elastic phase field approach. Soft Matter.

[48] Chaudhuri O., Gu L., Klumpers D., Darnell M., Bencherif S.A., Weaver J.C., Huebsch N., Lee H.-P., Lippens E., Duda G.N. (2016). Hydrogels with tunable stress relaxation regulate stem cell fate and activity. Nat. Mater..

[49] Engler A.J., Sen S., Sweeney H.L., Discher D.E. (2006). Matrix elasticity directs stem cell lineage specification. Cell.

[50] Zimmerling A., Yazdanpanah Z., Cooper D.M.L., Johnston J.D., Chen X. (2021). 3D printing PCL/nHA bone scaffolds: Exploring the influence of material synthesis techniques. Biomater. Res..

[51] Andreu I., Granero-Moya I., Garcia-Manyes S., Roca-Cusachs P. (2022). Understanding the role of mechanics in nucleocytoplasmic transport. APL Bioeng..

[52] Kelly D.J., Prendergast P.J., Blayney A.W. (2003). The effect of prosthesis design on vibration of the reconstructed ossicular chain: A comparative finite element analysis of four prostheses. Otol. Neurotol..

[53] Varga P., Willie B.M., Stephan C., Kozloff K.M., Zysset P.K. (2020). Finite element analysis of bone strength in osteogenesis imperfecta. Bone.

[54] Yang Z., Wang C., Gao H., Jia L., Zeng H., Zheng L., Wang C., Zhang H., Wang L., Song J. (2022). Biomechanical Effects of 3D-Printed Bioceramic Scaffolds with Porous Gradient Structures on the Regeneration of Alveolar Bone Defect: A Comprehensive Study. Front. Bioeng. Biotechnol..

[55] Patel S., Caldwell J., Doty S.B., Levine W.N., Rodeo S., Soslowsky L.J., Thomopoulos S., Lu H.H. (2018). Integrating soft and hard tissues via interface tissue engineering. J. Orthop. Res..

[56] Liao C., Li Y., Tjong S.C. (2020). Polyetheretherketone and Its Composites for Bone Replacement and Regeneration. Polymers.

[57] Liu Y.Y., Blazquez J.P.F., Yin G.Z., Wang D.Y., Llorca J., Echeverry-Rendón M. (2023). A strategy to tailor the mechanical and degradation properties of PCL-PEG-PCL based copolymers for biomedical application. Eur. Polym. J..

[58] Hutmacher D.W. (2000). Scaffolds in tissue engineering bone and cartilage. Biomaterials.

[59] Gao B., Yang Q., Zhao X., Jin G., Ma Y., Xu F. (2016). 4D Bioprinting for Biomedical Applications. Trends Biotechnol..

[60] Wan Z., Zhang P., Liu Y., Lv L., Zhou Y. (2020). Four-dimensional bioprinting: Current developments and applications in bone tissue engineering. Acta Biomater..

[61] Percival K.M., Paul V., Husseini G.A. (2024). Recent Advancements in Bone Tissue Engineering: Integrating Smart Scaffold Technologies and Bio-Responsive Systems for Enhanced Regeneration. Int. J. Mol. Sci..

[62] Xing Y., Qiu L., Liu D., Dai S., Sheu C.L. (2023). The role of smart polymeric biomaterials in bone regeneration: A review. Front. Bioeng. Biotechnol..

[63] Pobloth A.-M., Checa S., Razi H., Petersen A., Weaver J.C., Schmidt-Bleek K., Windolf M., Tatai A.Á., Roth C.P., Schaser K.-D. (2018). Mechanobiologically optimized 3D titanium-mesh scaffolds enhance bone regeneration in critical segmental defects in sheep. Sci. Transl. Med..

[64] Perren S.M. (2002). Evolution of the internal fixation of long bone fractures. The scientific basis of biological internal fixation: Choosing a new balance between stability and biology. J. Bone Jt. Surg. Br..

[65] Grunheid T., Langenbach G.E.J., Korfage J.A.M., Zentner A., van Eijden T.M.G.J. (2009). The adaptive response of jaw muscles to varying functional demands. Eur. J. Orthod..

[66] Safavi S., Yu Y., Robinson D.L., Gray H.A., Ackland D.C., Lee P.V.S. (2023). Additively manufactured controlled porous orthopedic joint replacement designs to reduce bone stress shielding: A systematic review. J. Orthop. Surg. Res..

[67] Tanveer M., Klein K., von Rechenberg B., Darwiche S., Dailey H.L. (2025). Don’t mind the gap: Reframing the Perren strain rule for fracture healing using insights from virtual mechanical testing. Bone Jt. Res..

[68] Claes L., Recknagel S., Ignatius A. (2012). Fracture healing under healthy and inflammatory conditions. Nat. Rev. Rheumatol..

[69] Glatt V., Evans C.H., Tetsworth K. (2016). A Concert between Biology and Biomechanics: The Influence of the Mechanical Environment on Bone Healing. Front. Physiol..

[70] Kadirvelu L., Sivaramalingam S.S., Jothivel D., Chithiraiselvan D.D., Karaiyagowder Govindarajan D., Kandaswamy K. (2024). A review on antimicrobial strategies in mitigating biofilm-associated infections on medical implants. Curr. Res. Microb. Sci..

[71] Chouirfa H., Bouloussa H., Migonney V., Falentin-Daudré C. (2019). Review of titanium surface modification techniques and coatings for antibacterial applications. Acta Biomater..

[72] Wei H., Cui J., Lin K., Xie J., Wang X. (2022). Recent advances in smart stimuli-responsive biomaterials for bone therapeutics and regeneration. Bone Res..

[73] Jiang C., Zhu G., Liu Q. (2024). Current application and future perspectives of antimicrobial degradable bone substitutes for chronic osteomyelitis. Front. Bioeng. Biotechnol..

[74] Rana M.M., De la Hoz Siegler H. (2024). Evolution of Hybrid Hydrogels: Next-Generation Biomaterials for Drug Delivery and Tissue Engineering. Gels.

[75] Elsalanty M.E., Genecov D.G. (2009). Bone grafts in craniofacial surgery. Craniomaxillofac. Trauma Reconstr..

[76] Min S., Ko I.K., Yoo J.J. (2019). State-of-the-Art Strategies for the Vascularization of Three-Dimensional Engineered Organs. Vasc. Spec. Int..

[77] Ghanaati S., Śmieszek-Wilczewska J., Al-Maawi S., Neff P., Zadeh H.H., Sader R., Heselich A., Rutkowski J.L. (2022). Solid PRF Serves as Basis for Guided Open Wound Healing of the Ridge after Tooth Extraction by Accelerating the Wound Healing Time Course-A Prospective Parallel Arm Randomized Controlled Single Blind Trial. Bioengineering.

[78] Liu P., Tu J., Wang W., Li Z., Li Y., Yu X., Zhang Z. (2022). Effects of Mechanical Stress Stimulation on Function and Expression Mechanism of Osteoblasts. Front. Bioeng. Biotechnol..

[79] Dérand P., Rännar L.E., Hirsch J.M. (2012). Imaging, virtual planning, design, and production of patient-specific implants and clinical validation in craniomaxillofacial surgery. Craniomaxillofac. Trauma Reconstr..

[80] Kudva A., Thomas J., Saha M., Srikanth G., Kamath A.T., Abhijith S.M. (2025). Mandibular Reconstruction Modalities Using Virtual Surgical Planning and 3D Printing Technology: A Tertiary Care Centre Experience. J. Maxillofac. Oral Surg..

[81] Ghiasi M.S., Chen J., Vaziri A., Rodriguez E.K., Nazarian A. (2017). Bone fracture healing in mechanobiological modeling: A review of principles and methods. Bone Rep..

